# The Use of Plant-Derived Ribosome Inactivating Proteins in Immunotoxin Development: Past, Present and Future Generations

**DOI:** 10.3390/toxins9110344

**Published:** 2017-10-27

**Authors:** Aleksander Rust, Lynda J. Partridge, Bazbek Davletov, Guillaume M. Hautbergue

**Affiliations:** 1 Structural and Molecular Biology, Division of Biosciences, Faculty of Life Sciences, University College London, London WC1E 6BT, UK; 2 Department of Molecular Biology and Biotechnology, University of Sheffield, Firth Court, Western Bank, Sheffield S10 2TN, UK; l.partridge@sheffield.ac.uk; 3 Department of Biomedical Science, University of Sheffield, Firth Court, Western Bank, Sheffield S10 2TN, UK; b.davletov@sheffield.ac.uk; 4 Sheffield Institute for Translational Neuroscience, Department of Neuroscience, University of Sheffield, 385a Glossop Road, Sheffield S10 2HQ, UK

**Keywords:** ribosome inactivating proteins, immunotoxins, therapeutic applications

## Abstract

Ribosome inactivating proteins (RIPs) form a class of toxins that was identified over a century ago. They continue to fascinate scientists and the public due to their very high activity and long-term stability which might find useful applications in the therapeutic killing of unwanted cells but can also be used in acts of terror. We will focus our review on the canonical plant-derived RIPs which display ribosomal RNA *N*-glycosidase activity and irreversibly inhibit protein synthesis by cleaving the 28S ribosomal RNA of the large 60S subunit of eukaryotic ribosomes. We will place particular emphasis on therapeutic applications and the generation of immunotoxins by coupling antibodies to RIPs in an attempt to target specific cells. Several generations of immunotoxins have been developed and we will review their optimisation as well as their use and limitations in pre-clinical and clinical trials. Finally, we endeavour to provide a perspective on potential future developments for the therapeutic use of immunotoxins.

## 1. Introduction

Ribosome inactivating proteins (RIPs) are a family of toxins that irreversibly inhibit eukaryotic protein synthesis [1,2,3]. Although most commonly found in plants, RIPs have also been identified in bacteria, fungi and even in two mosquito species [4]. RIPs form a heterogeneous group of proteins with varied enzyme functionalities and several classifications have been proposed [1,5]. This review will focus on the canonical plant-derived RIPs as they are the most characterised and commonly used toxins in immunotoxin development. In particular, we will review how these RIPs have been utilised in the potential treatment of cancer which currently holds the most promising applications for immunotoxin therapy.

Purification of the RIP ricin and subsequent immunological experiments carried out by Paul Ehrlich in the early 19th century led to the concept of the ‘magic bullet’ [2]. This is the idea of an exceptionally toxic molecule that can specifically attack and kill target cells [6]. It became a highly attractive concept for the treatment of cancer and was built upon in the 1970s when the first immunotoxins—a protein synthesis inhibiting toxin conjugated to a targeting antibody—were generated [7]. As recombinant technology has started to dominate immunotoxin design incorporating bacterial toxic enzymes, the use of plant RIPs has recently decreased; however, these toxins still display a number of characteristics that make them attractive in immunotoxin technology. Moreover, pre-clinical studies using plant RIPs are showing promise for their use in future therapy.

## 2. Ribosome Inactivating Proteins with Ribosomal RNA *N*-Glycosidase Activity

RIPs are found almost ubiquitously among plants and are thought to act as a form of immune defence, as upregulation of expression can be seen following viral infection and contamination with microorganisms [8] as well as abiotic stress [9]. RIPs are renowned active substances that have been used in traditional Chinese medicine for centuries. Trichosanthin is one typical example that shows promising outcomes in the killing of cancer cells, particularly hepatocellular carcinoma, both in vitro and in vivo in a murine xenograft model [10]. There are currently almost 250 proteins that irreversibly inactivate protein synthesis. They can be divided into several broad groups with a two-category classification (type 1 and 2) prevailing (reviewed in [1,2,5]). Type 1 RIPs are monomeric proteins of approximately 30 kDa with enzymatic activity, and type 2 RIPs are heterodimeric proteins that contain an enzymatic domain of approximately 30 kDa (A-chain) linked via a disulphide bond to a second lectin-like domain of approximately 35 kDa (B-chain) which is able to bind to cells and facilitate internalisation [11].

Type 2 RIPs such as ricin are able to bind to sugars on the cell surface via their lectin-like B-chain. Although this means that type 2 RIPs are generally more potent than type 1 RIPs, cell binding alone is not sufficient to confer potency, as toxicity can vary greatly between type 2 RIPs—some RIPs being considered non-toxic. For instance, Ricinus agglutinin (RCA) and ricin are both type 2 RIPs found in castor beans, but ricin shows around 68-fold higher potency than RCA in cells, likely due to a decreased ability of RCA to translocate into the cytoplasm [11]. Similarly, some type 2 RIPs isolated from some *Sambucus* species, such as nigrins or ebulins, exhibit high ribosome-inactivating activities in cell-free systems but lack toxicity both in vitro and in vivo. This is because these RIPs follow a different intracellular trafficking route with the majority of molecules being either recycled to the cell surface or degraded [12,13]. These examples highlight the importance of intracellular trafficking following binding for mediating cytotoxicity. Studies using ricin show that, following binding, the toxin is taken up by both clathrin-dependent and -independent endocytosis and a small percentage localises with the trans-Golgi network, followed by retrograde transport to the ER [14]. Once in the lumen of the ER, it is thought that the A-chain is cleaved from the B-chain by the protein disulphide isomerase and is then processed by the ER as a misfolded protein, meaning that it is exported to the cytosol for degradation [15,16]. Upon entering the cytosol, the A-chain is refolded by the sequential utilisation of the Hsc70 and Hsp90 chaperone systems and the correctly folded native A-chain is then able to carry out its catalytic activity at the ribosomes [17].

Type 1 RIPs such as saporin and gelonin lack the cell-binding B-chain of type 2 RIPs and are therefore much less cytotoxic than most type 2 RIPs. It is thought that uptake generally occurs through a passive manner, such as by fluid-phase pinocytosis [18]. It has also been proposed that saporin can enter cells in a receptor-dependent manner, via binding to α2-macroglobulin receptors [19]. However, similar sensitivities to saporin have been observed between α2-macroglobulin receptor expressing and non-expressing cell lines which would indicate that saporin internalisation does not occur via this receptor [20]. The mechanism of endocytosis of type 1 RIPs remains unclear, but studies with saporin appear to show an internalisation mechanism that is independent of the Golgi apparatus, suggesting that it follows a distinct pathway to ricin [21]. Nevertheless, upon reaching the cytosol, many type 1 RIPs display a highly active enzymatic action, and artificial delivery into the cell or attachment to a targeting ligand leads to cytotoxicity with high potency [11,22].

Types 1 and 2 RIPs display ribosomal RNA *N*-glycosidase activity (EC 3.2.2.22) and cleave the 28S rRNA by removal of a single adenine residue (A4324 in rat rRNA) from a GAGA sequence at the universally conserved α-sarcin/ricin loop [1,2,5]. This further prevents the recruitment of eukaryotic elongation translation factors eEF1/2 to the 60S ribosomal subunit and translocation of the ribosome and protein synthesis [23,24,25] thus causing a complete and irreversible block of protein synthesis [26]. It was initially thought that cells underwent apoptosis after exposure to RIPs solely due to the ribotoxic stress response after inhibition of protein synthesis. However, more recent data suggests that RIPs may also exhibit other activities independently of their targeting of protein synthesis. For instance, it has been shown that RIPs show adenine glycosidase activity in DNA, RNA and poly (A) [27]. Additionally, ricin has been shown to cause early nuclear DNA damage independently of protein synthesis inhibition, and saporin S6 was shown to induce apoptosis through mitochondrial cascade prior to the onset of protein synthesis inhibition [28,29]. It has therefore been proposed that RIPs may induce apoptosis by a number of different mechanisms, of which inhibition of protein synthesis plays an important but not always essential role [30].

## 3. The Development of RIP-Based Immunotoxins

A common feature of many RIPs is their extraordinarily high level of potency. As with other protein synthesis inhibiting toxins, such as diphtheria toxin, it is thought that only a few molecules are needed to enter the cytosol of a cell for cell death to occur. This potency makes them highly attractive as a possible cancer therapeutic. However, this is a double-edged sword as toxicity is achieved in both healthy and malignant cells, meaning that these toxins must be efficiently targeted to cancer cells to convey specific anticancer activity. The main methods by which toxins are targeted to cancer cells are either by conjugation to an antibody to make an immunotoxin, or to a targeting ligand such as a growth factor or cytokine [31]. Immunotoxins are becoming the predominant choice as they allow for greater selectivity and flexibility when choosing a target. Selecting an appropriate target is of high importance when generating a targeted toxin, as it has a large impact on specificity and potency. The chosen target must be highly expressed on the surface of the cancer cell, but have relatively restricted expression in healthy tissue, as this limits on-target off-tumour toxicity [32]. For immunotoxins, this generally means targeting to tumour-associated antigens, which are highly expressed on the cell surface as a result of transformation [33]. Since the generation of the first immunotoxins in the 1970s, more than 450 immunotoxins have been used considering RIPs alone [31]. The most common plant-based RIPs used in immunotoxin development are the chain A of the type 2 RIP ricin as well as type 1 RIPs saporin and gelonin which all exhibit high potency and stability. It has also been recently suggested to use type 2 RIPs with low in vitro and in vivo toxicity but potent ribosomal RNA *N*-glycosidase activities in cell-free systems, such as the chains A of ebulins and nigrins from the *Sambucus* species, to generate alternative immunotoxins with very high cytotoxicity [12,13].

As antibody therapy and recombinant technology has advanced, so too has immunotoxin design, progressing from simple chemical conjugation of a native toxin to a whole antibody, to the recombinant engineering of modified toxin domains fused with humanised antibody fragments. The progression of development can be broadly split into three generations.

### 3.1. First-Generation Immunotoxins

**Figure 1:**
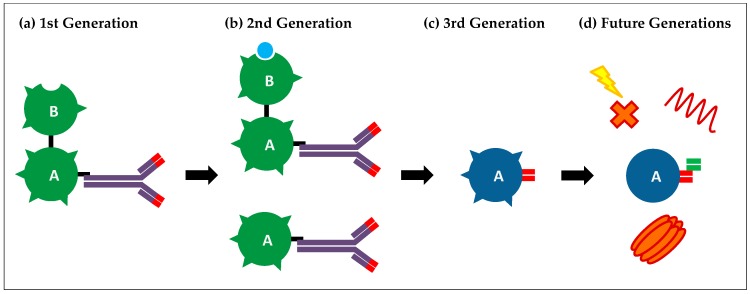
Diagram depicting the different generations of immunotoxins. (**a**) First-generation immunotoxins. Purified toxins were chemically linked to a targeting antibody; (**b**) Second-generation immunotoxins. Purified type 1 ribosome inactivating proteins (RIPs) or type 2 RIPs with B-chain either blocked or removed were chemically linked to a targeting antibody; (**c**) Third-generation immunotoxins. Recombinant purified toxins were fused to antibody targeting fragments; (**d**) Future generation immunotoxins. Toxins are modified to remove immunogenic epitopes and exhibit dual targeting abilities to improve specificity. They can be co-administered with endosome disruptive agents, such as pore-forming agents, endosome disruptive peptides, or photosensitisers, to increase intracellular delivery and potency.

The first-generation immunotoxins were developed in the early 1970s and were usually made using a full toxin chemically linked to a whole monoclonal antibody (Figure 1a).

Initial studies with first-generation immunotoxins were primarily carried out with the diphtheria toxin: a bacterial toxin that is analogous to type 2 RIPs in that it has two distinct domains for targeting and enzymatic action to inhibit protein synthesis [7,34,35]. Although these often gave promising results in vitro, a number of issues were encountered upon testing in vivo. The major drawback of these immunotoxins was the presence of the targeting domain, which meant that the protein was able to bind to and enter a wide range of different cells, irrespective of target antigen expression. This caused a high level of non-specific toxicity and intolerable side effects. At around this time the type 2 RIPs ricin and abrin were gaining attention as anti-cancer agents as they were found to more efficiently inhibit protein synthesis in certain tumour models than in healthy cells. Additionally, these unmodified toxins were shown to have anti-tumour properties in Ehrlich ascites tumour mouse models [36]. However, non-specific toxicity was still an issue with only very low doses needed to cause lethality [37]. Re-targeting of RIPs to cancer cells was therefore necessary, and they were increasingly used in the design of second-generation immunotoxins.

Despite a large number of issues with these original toxins, impressive in vitro data displayed the potential of immunotoxins for cancer treatment and a large amount of effort was applied to increase effectiveness in vivo.

### 3.2. Second-Generation Immunotoxins

A greater understanding of toxin structure and function led to the second generation of immunotoxins in the mid-1970s. These usually consisted of a toxin lacking a cell-binding domain, which greatly reduced non-specific internalisation, allowing for the administration of higher doses and a greater therapeutic window (Figure 1b). The use of RIPs was, and still is, popular for this generation of immunotoxins as purification techniques are well established and they are highly stable proteins. Ricin-based immunotoxins of the second generation were produced in 1976 and consisted of the castor bean ricin A-chain chemically linked to cancer-targeting antibodies [38]. By the mid-1980s, a number of ricin A-chain conjugates had been developed which demonstrated efficacy against various cancer models in vitro including leukaemia and breast cancer, as well as showing efficacy in vivo [39].

In these second-generation toxins, the A-chain was isolated by purification of the complete protein followed by removal of the B-chain by reduction of the disulphide bond that joins the two domains. This made purification of utmost importance as, due to the high potency of native ricin, any residual full toxin would cause unwanted side effects. A second issue with the A-chain is that it is glycosylated, meaning that it can bind to and be internalised by cells expressing appropriate mannose receptors, leading to reduced stability in blood circulation and residual non-specific toxicity [40]. The A-chain therefore needed to be deglycosylated before use in immunotoxins, further complicating production. An alternative method was to use the entire ricin toxin with the B-chain blocked by chemical modification or the addition of lactose (Figure 1b). These conjugates often showed higher in vitro potency than A-chain only conjugates, and it was postulated that this was due to the B-chain facilitating entry into the cell cytosol [41], or unblocking of the B-chain upon cell binding, allowing for increased cellular interaction with galactose binding sites [42]. However, these blocked-ricin immunotoxins also demonstrated higher non-specific toxicity than their “A-chain only” counterparts.

The first type 1 RIP-based immunotoxin was generated in 1981 and consisted of gelonin conjugated to an anti-Thy1.1 antibody [43]. This showed high cytotoxicity in vitro in lymphoma cell lines and significantly prolonged the life of mice bearing lymphoma allografts. Pokeweed antiviral protein (PAP), another type 1 RIP, was also used in immunotoxin generation and showed similar potency against Thy1.1 positive cells when directly compared to a ricin A-chain immunotoxin [44]. Type 1 RIPs have an advantage over type 2 RIPs in that they natively lack a cell-binding domain, so further modification of the toxin is not necessary before antibody conjugation. Since then, saporin has become the more popular type 1 RIP for immunotoxin development due to its high thermodynamic stability [2].

The second generation of immunotoxins also saw the use of recombinant technology for toxin production and the ricin A-chain was expressed in *Escherichia coli* in 1987 [45]. Despite issues expressing plant-based toxins, recombinant expression holds a number of advantages over traditional purification methods. Firstly, it is a much more straightforward process and does not require complex and lengthy procedures to eliminate the residual B-chain. Secondly it is much safer, as no highly potent full toxin needs to be handled. Finally, prokaryotic expression means that the protein is not glycosylated, helping to prolong toxin stability and reduce toxicity in vivo. A number of type 1 RIPs, including saporin and gelonin, have also been generated recombinantly [46,47].

More promising in vivo data obtained from second-generation immunotoxins led to the first clinical trial in 1986 [48]. This trial used the ricin A-chain linked to a pan-T-lymphocyte antibody (T101) in the treatment of two leukaemia patients, and showed promising results with large drops in target cell numbers. Follow-up phase I and II trials also demonstrated efficacy, but revealed a number of dose-limiting side effects including a drop in serum albumin levels, weight gain, and oedema, caused by off-target toxicity of the immunotoxin [49]. The first clinical trial with a type 1 RIP-based immunotoxin was carried out in 1992 using a saporin-anti-CD30 conjugate for the treatment of Hodgkin’s disease. It successfully led to a 50 to 75% reduction of the tumour mass in 3 out of 4 patients, but also caused side effects such as oedema [50]. Similar dose-limiting side effects were seen in a number of other immunotoxin clinical trials [51,52] and this was later attributed to vascular leak syndrome (VLS) [53]. VLS occurs when non-specific uptake of the toxin into endothelial cells causes cell death and leakage of fluid from the circulatory system into the interstitial space, leading to oedema, weight gain, hypoalbuminemia, pulmonary infiltrates, and hypotension [54].

As well as dose-limiting toxicity, further problems persisted in second-generation immunotoxins. As with the first generation, second-generation immunotoxins were chemically linked to targeting antibodies through a disulphide bond so that, upon entry into endosomes, an increase in reducing conditions breaks the bond and frees the toxin. However, premature nucleophilic attack of the disulphide bond, particularly in the anaerobic reducing environment of tumours, caused low stability in vivo [55]. Inefficient chemical linking also led to heterogeneous compositions of bound and unbound components which could interfere with antigen binding [56]. As well as instability and non-specific binding, these conjugates were also very large (180–200 kDa) which affected tumour penetration [57]. This is why the most promising results were seen in haematological cancers rather than solid tumours. Another factor affecting tumour penetration was the short half-life, which was around 30 min in native ricin A-chain immunotoxins. This issue was addressed by de-glycosylation of the toxin which helped to increase circulating half-life to 4–6 h [57]. Longer treatment periods and higher concentrations unveiled a further problem with immunotoxins, which is immunogenicity [58]. Both the antibody and toxin were obtained from non-human sources—mice and plants respectively—meaning that they contained many immunogenic epitopes. Repeated exposure would therefore elicit an immune response, negating the anti-tumour effect of the immunotoxin.

The second generation of immunotoxins showed large improvements over the first generation, allowing higher tolerance in animals and the first clinical trials. However, many problems persisted that limited the therapeutic benefit of this treatment. Advances in recombinant technology allowed for greater control over design and production of immunotoxins, so that a number of these issues could be addressed.

### 3.3. Third-Generation Immunotoxins

Third-generation immunotoxins utilise advanced genetic engineering techniques to generate fully recombinant toxins fused to the targeting domain of a monoclonal antibody [59] (Figure 1c). These are commonly known as recombinant immunotoxins (RITs). Most RITs are generated from bacterial toxins such as diphtheria toxin or pseudomonas exotoxin A, as these are easier to express in bacteria. A RIT consisting of the enzymatic and translocation domains of pseudomonas exotoxin A fused to the heavy and light chain portions of a monoclonal antibody targeting B3 was reported in 1995 [60]. This RIT demonstrated selective cytotoxicity in vitro and in vivo that was several-fold higher than an equivalent, chemically linked immunotoxin. The main advantage of this approach is that a homogenous population is produced which gives increased stability and reduced interference of antigen binding. This approach also allows the incorporation of more stable linkers that are cleavable only by intracellular enzymes. For instance, furin-sensitive linker sequences between the targeting and enzymatic domains have shown significant improvements in toxicity of pseudomonas exotoxin A immunotoxins [61].

The use of antibody fragments allowed the generation of immunotoxins of much smaller sizes. The smallest antibody fragments that retain targeting ability consist of one variable heavy chain and one variable light chain (scFv) and allow for the generation of immunotoxins as small as 60 kDa [56]. Although these showed increased tumour penetration, they were rapidly cleared from the blood (circulating half-life of approximately 20 min) leading to decreased therapeutic efficacy. Another issue with smaller RITs is that they are cleared by renal filtration and can accumulate in the kidney, leading to renal toxicity [62]. It is therefore thought that RITs should be over 65 kDa in size as this is the cut-off limit for macromolecules cleared by renal filtration [63]. Thus, a balance needs to be found between decreasing size to increase tumour penetration, without affecting plasma half-life. Another issue with the use of a single scFv antibody portion is instability, as this lacks the stabilising disulphide bond found in the absent portion [64]. As a result of this, the heavy and light chains can dissociate and bind to other dissociated chains leading to aggregation and loss of activity. However, further engineering introduced a disulphide bond into the Fv domain which prevented dissociation without affecting binding affinity [65]. Another alternative is the use of nanobodies, monomeric and variable heavy chain antibodies that occur naturally in the *Camelidae* family [66]. These offer a number of advantages over conventional antibodies, including their small size, low immunogenicity, high stability, and ease of production. They have been recently used successfully in the development of a number of immunotoxins [67,68,69].

Bacterial toxins are much more common in fusion immunotoxins as they are easier to express in bacterial systems. Type 1 RIP-based fusion proteins have been successfully produced in bacteria [70,71], but are less common as they often suffer from low stability and activity due to incorrect folding. However, it has been demonstrated that the yeast *Pichia pastoris* may be used for RIT generation [72], and a recent study has shown that saporin-antibody fusions generated using this system demonstrate comparable in vitro potency to bacterial-based immunotoxins [73]. Yeast may be a more suitable system for RIT production as they are eukaryotic and therefore capable of the complex folding and post-translational modifications necessary to generate functional RITs. A drawback is that they are eukaryotic and therefore susceptible to the cytotoxic mechanism of the toxin. However, certain strains of *Pichia pastoris* (e.g., GS115) have displayed a high level of resistance to toxic action which is attributed to the rapid secretion of the protein into the culture medium [72].

Due to the issues with generating recombinant RIP-based immunotoxins, the vast majority of clinical trials have been, or are being, carried out with bacterial-based immunotoxins, particularly pseudomonas exotoxin A. Indeed, most recent trials using ricin are based on deglycosylated A-chain or blocked-ricin second-generation immunotoxins [31,74,75,76]. Two earlier phase I trials using recombinant ricin were carried out in breast cancer patients, but significant off-target toxicity was still observed in both variants [77,78]. Phase I clinical trials have also been carried out using the recombinantly generated type 1 RIPs gelonin and bouganin. VB6-845 is a recombinant fusion protein consisting of modified bouganin and an anti-epithelial cell adhesion molecule (epCAM) Fab moiety which was tested in a phase I trial for treating epithelial tumours (Clinicaltrials.gov identifier: NCT00481936). However, results from this trial remain unpublished. Recombinant gelonin conjugated to a humanised anti-CD33 antibody (M195) has also been tested in a phase I trial for the treatment of refractory myeloid leukaemia [79]. Although low response rates were reported, this conjugate nevertheless exhibited promising characteristics when compared to bacterial immunotoxins, including low immunogenicity and an absence of VLS as a side effect.

## 4. Current Limitations and Future Generations of Immunotoxins

Advances in antibody therapy and recombinant technology have sparked a resurgence in interest for targeted toxins as a possible therapeutic approach, and a number of promising results have been obtained for the treatment of haematological malignancies. However, issues still need to be addressed to make these therapies more effective, particularly in solid tumours, including increasing bioavailability and tumour penetration, reducing immunogenicity and reducing off-target toxicity. Indeed, due to these issues, only one targeted toxin, denileukin diftitox, has FDA approval for cancer therapy in humans. Denileukin diftitox (also known as Ontak) is a targeted toxin made up of the enzymatic and translocation domains of *Diphtheria* toxin recombinantly fused to IL-2 used in the treatment of lymphoma [80]. The most successful application of a type 1 RIP-based targeted toxin is the use of recombinant saporin linked to Substance P for the treatment of bone cancer pain in old dogs [81,82]. The FDA has already approved Minor Use/Minor Species (MUMS) designation for this drug providing extended market exclusivity to treat the >10,000 annual cases of canine bone cancer-related pain.

Numerous groups are working on the inherent problems, and incorporation of their findings into immunotoxin design may drastically improve the efficacy of future generations. For example, an antibody can target the toxins only to a specific subset of cells, meaning that any heterogeneity within the tumour with regards to antigen expression will lead to resistant cancer cells. To overcome this, immunotoxins that can simultaneously target different antigens are being tested (Figure 1d). Combotox is the co-administration of ricin A-chain-based immunotoxins such as those that target CD19 and CD22 to treat B-lineage lymphoblastic leukaemia. This treatment has shown efficacy in phase I clinical trials [74]. A recent study demonstrated that the use of antibodies targeting the Neuron-glia 2 (NG2) or Ganglioside D3 (GD3) antigens can greatly increase the efficacy of saporin immunotoxins in in vitro models of glioblastoma [83]. NG2 and GD3 are respectively associated with two distinct cell sub-populations, fast dividing NG2 positive cells and GD3 positive cells that are involved in survival and migration. Targeting known sub-populations within a cancer will help to improve response rates and decrease relapse. As well as the co-administration of immunotoxins, the use of bi-specific antibodies allows one molecule to target multiple antigens. For instance, tandem scFv segments have been successfully used to target CD19 and CD22 in both pseudomonas exotoxin A and diphtheria toxin immunotoxins. These fusions have shown increased efficacy in B-cell lymphoma mice models when compared with immunotoxins targeting just one antigen [84,85]. As well as helping to overcome tumour-heterogeneity, dual-antigen targeting may help to decrease on-target, off-tumour toxicity caused by low-level antigen expression on healthy cells. Antigens found exclusively on cancer cells are rare which severely limits targeting choices [63]. Indeed, unexpected, low-level expression of a target antigen in certain tissues has limited the use of a number of immunotoxins [32]. The selection of two TAAs expressed on a cancer cell surface will help to increase specific tumour cell binding and reduce internalisation into healthy cells.

Off-target toxicity due to non-specific uptake of toxin leads to dose-limiting side effects such as VLS, hepatotoxicity, renal toxicity, and cardiac dysfunction. As mentioned previously, VLS was observed as a side effect in the first clinical trials and has continued to affect all immunotoxin treatments tested to date. It is particularly severe in ricin-based therapies, even resulting in fatalities in some trials [63,86]. One study identified short amino-acid motifs (x)-D-(y) (where x is L, I, G or V and y is V, L or S) which may bind to endothelial cells resulting in internalisation of the toxin and cell death [87]. Modification of these motifs in ricin led to the generation of a toxin with reduced ability to cause vascular leak syndrome in mice [88]. Pre-treatment of patients with steroids to reduce inflammatory responses has also been shown to help combat VLS [89].

Improved cytosolic delivery of the toxin would also help to reduce side effects, as lower doses would need to be administered to observe the same therapeutic effect. Additionally, more efficient cytosolic entry may give increased efficacy in solid tumours where only a small number of molecules reach target cells. It is thought that only a small percentage of internalised toxin is able to evade lysosomal degradation and enter the cytosol. Indeed, a study using targeted gelonin found a near-universal requirement of 5 million molecules needed to be internalised for cell killing, despite different routes of binding and internalisation [90]. Considering the high potency of RIPs with in vitro turnover of 28S rRNA depurination of 700–800 molecules/min, this strongly suggests that escape from the endolysosomal compartment is the rate-limiting step that determines efficacy. Various methods are in development to improve endosome escape, including the co-administration of endosome disrupting agents (Figure 1d). For instance, one study found that co-administration of targeted gelonin with listeriolysin targeted to the same antigen led to a large increase in efficacy and was well tolerated in vivo [91]. Lysteriolysin is a cytolysin protein produced by the bacterium *Listeria monocytogenes* which can lyse endosomes in a pH-dependent manner. Co-administration of plant-based saponins, glycosides that can form pores in membranes, has also been found to increase potency whilst maintaining target specificity of targeted toxins [92]. A range of endosome escape peptides are available which are able to disrupt membranes in a pH-dependent manner. For instance, the GALA peptide is a short, 30-residue, synthetic peptide with a repeating Glu-Ala-Leu-Ala sequence [93]. GALA mimics the function of viral fusion protein segments that mediate the escape of viral genes from endosomes into the cytosol. Endosomal acidification causes a rearrangement of the peptide structure from random to helical, giving it a high affinity for neutral or negatively charged membranes, leading to the formation of pores and destabilisation of the endosomal membrane [93]. An alternative method is the use of photochemical internalisation which utilises an endocytic vesicle-localising photosensitiser that generates reactive oxygen species upon exposure to light, triggering vesicle rupture [94]. Preferential retention of the sensitiser in tumour cells and focused light application using a laser adds a degree of selectivity to this technique which, combined with specific targeting by the toxin, can reduce side effects and increase the therapeutic window [95]. Photochemical internalisation has been used successfully in vivo to enhance the efficacy of a saporin-based immunotoxin that targets the cancer stem cell marker CD133 [96].

Development of humanised antibodies by combining the recognition domain with human framework regions has largely stopped immune reactions to the targeting moiety of immunotoxins [97], but problems persist with the toxins themselves. Methods employed to reduce immunogenicity of the toxins include chemical modification and removal of immunogenic epitopes. Chemical modification with polyethene glycol (PEGylation) is a common technique used to increase the plasma half-life of therapeutic proteins [98]. Moreover, site-specific PEGylation of an IL-2 targeting pseudomonas exotoxin A based immunotoxin was found to dramatically reduce immunogenicity in mice, and was thought to act by reducing protein degradation in antigen-presenting cells as well as shielding some epitopes following degradation [99]. Immunogenicity of toxins can be greatly reduced by the removal of immunogenic epitopes. In one study, the antigenic domains of gelonin were successfully mapped and deleted to create smaller, modified toxins that retained enzymatic activity but exhibited reduced antigenicity in vitro [100]. T-cell recognition epitopes were identified and mutated in the type I RIP bouganin to create an epitope-depleted mutant de-bouganin [101]. An immunotoxin based on de-bouganin has been shown to be well tolerated in vivo with minimal side effects and low immunogenicity [102]. B-cell epitopes have been successfully removed from pseudomonas exotoxin A by isolating antibodies from patients with immune resistance to this toxin and constructing a phage display library [103]. Alanine scanning mutagenesis was then used to locate the epitopes and an alternative toxin was generated (LR-O10) which showed low reactivity with human antisera but maintained high cytotoxic and anti-tumour activity. Another possibility is to utilise the wide range of plant-based RIPs that are available. These RIPs are often immunogenically distinct and so development of a treatment regimen that utilises a combination of immunotoxins containing different RIPs may help to prolong the number of treatment rounds whilst avoiding an immune response.

An additional way to improve the success of immunotoxin therapy may be to alter how these proteins are utilised. The majority of trials for solid cancers are carried out in patients with advanced disease and high tumour burden. A more effective strategy may be the use of immunotoxins following traditional chemotherapy to clear up minimal residual disease. This is the small number of cells that are often resistant to chemotherapy and remain circulating in the body with the ability to cause relapse [104]. This would be a more suitable target for immunotoxins as issues with tumour penetration would be bypassed. Immunotoxins have been thought to be particularly applicable to bladder cancer as this organ could be thought of as an “external” environment that would restrict the immune reactions and side effects caused by immunotoxins [105,106]. Moreover, it was recently reported that a complete and at least a 3 year long-lasting elimination of bladder cancer was observed following treatment with an immunotoxin prepared with the ricin A-chain [107].

## 5. Perspectives

A number of advances have been made in immunotoxin design which have taken them closer to clinical use as a therapy for a variety of cancers. RIPs have been instrumental in this advancement, from the original concept of the ‘magic bullet’ in the late 1800s to modern-day clinical trials. Recombinant technology has seen a rise in the use of bacterial toxins over RIPs. More recently, human cytotoxic enzymes such as granzyme B and ribonuclease have also been utilised in what has been called the fourth generation of immunotoxins [108,109,110]. However, numerous groups are still utilising plant-based RIPs to tackle current problems with these treatments including production [73], cell delivery [91,92] and immunogenicity [100,101]. These toxins are therefore useful tools for immunotoxin design and may yet be seen in future clinical trials.

## References

[1] Schrot J., Weng A., Melzig M.F. (2015). Ribosome-inactivating and related proteins. Toxins.

[2] Bolognesi A., Bortolotti M., Maiello S., Battelli M., Polito L. (2016). Ribosome-inactivating proteins from plants: A historical overview. Molecules.

[3] Fabbrini M.S., Katayama M., Nakase I., Vago R. (2017). Plant ribosome-inactivating proteins: Progesses, challenges and biotechnological applications (and a few digressions). Toxins.

[4] Lapadula W.J., Sanchez Puerta M.V., Juri Ayub M. (2013). Revising the taxonomic distribution, origin and evolution of ribosome inactivating protein genes. PLoS ONE.

[5] Walsh M.J., Dodd J.E., Hautbergue G.M. (2013). Ribosome-inactivating proteins: Potent poisons and molecular tools. Virulence.

[6] Antignani A., Fitzgerald D. (2013). Immunotoxins: The role of the toxin. Toxins.

[7] Moolten F.L., Cooperband S.R. (1970). Selective destruction of target cells by diphtheria toxin conjugated to antibody directed against antigens on the cells. Science.

[8] Wong R.N.S., Mak N.K., Choi W.T., Law P.T.W. (1995). Increased accumulation of trichosanthin in trichosanthes kirilowii induced by microorganisms. J. Exp. Bot..

[9] Rippmann J.F., Michalowski C.B., Nelson D.E., Bohnert H.J. (1997). Induction of a ribosome-inactivating protein upon environmental stress. Plant Mol. Biol..

[10] Zhang Y.H., Wang Y., Yusufali A.H., Ashby F., Zhang D., Yin Z.F., Aslanidi G.V., Srivastava A., Ling C.Q., Ling C. (2014). Cytotoxic genes from traditional chinese medicine inhibit tumor growth both in vitro and in vivo. J. Integr. Med..

[11] Stirpe F., Battelli M.G. (2006). Ribosome-inactivating proteins: Progress and problems. Cell. Mol. Life Sci..

[12] Ferreras J.M., Citores L., Iglesias R., Jimenez P., Girbes T. (2011). Use of ribosome-inactivating proteins from sambucus for the construction of immunotoxins and conjugates for cancer therapy. Toxins.

[13] Jimenez P., Tejero J., Cordoba-Diaz D., Quinto E.J., Garrosa M., Gayoso M.J., Girbes T. (2015). Ebulin from dwarf elder (*sambucus ebulus* L.): A mini-review. Toxins.

[14] Sandvig K., van Deurs B. (1996). Endocytosis, intracellular transport, and cytotoxic action of shiga toxin and ricin. Physiol. Rev..

[15] Roberts L.M., Lord J.M. (2004). Ribosome-inactivating proteins: Entry into mammalian cells and intracellular routing. Mini Rev. Med. Chem..

[16] Spooner R.A., Watson P.D., Marsden C.J., Smith D.C., Moore K.A., Cook J.P., Lord J.M., Roberts L.M. (2004). Protein disulphide-isomerase reduces ricin to its a and b chains in the endoplasmic reticulum. Biochem. J..

[17] Spooner R.A., Hart P.J., Cook J.P., Pietroni P., Rogon C., Hohfeld J., Roberts L.M., Lord J.M. (2008). Cytosolic chaperones influence the fate of a toxin dislocated from the endoplasmic reticulum. Proc. Natl. Acad. Sci. USA.

[18] Polito L., Bortolotti M., Mercatelli D., Battelli M.G., Bolognesi A. (2013). Saporin-s6: A useful tool in cancer therapy. Toxins.

[19] Cavallaro U., Nykjaer A., Nielsen M., Soria M.R. (1995). Alpha 2-macroglobulin receptor mediates binding and cytotoxicity of plant ribosome-inactivating proteins. Eur. J. Biochem..

[20] Bagga S., Hosur M.V., Batra J.K. (2003). Cytotoxicity of ribosome-inactivating protein saporin is not mediated through alpha2-macroglobulin receptor. FEBS Lett..

[21] Vago R., Marsden C.J., Lord J.M., Ippoliti R., Flavell D.J., Flavell S.U., Ceriotti A., Fabbrini M.S. (2005). Saporin and ricin a chain follow different intracellular routes to enter the cytosol of intoxicated cells. FEBS J..

[22] Rust A., Hassan H.H., Sedelnikova S., Niranjan D., Hautbergue G., Abbas S.A., Partridge L., Rice D., Binz T., Davletov B. (2015). Two complementary approaches for intracellular delivery of exogenous enzymes. Sci. Rep..

[23] Endo Y., Tsurugi K. (1987). Rna n-glycosidase activity of ricin a-chain. Mechanism of action of the toxic lectin ricin on eukaryotic ribosomes. J. Biol. Chem..

[24] Endo Y., Mitsui K., Motizuki M., Tsurugi K. (1987). The mechanism of action of ricin and related toxic lectins on eukaryotic ribosomes. The site and the characteristics of the modification in 28 s ribosomal rna caused by the toxins. J. Biol. Chem..

[25] Endo Y., Tsurugi K. (1988). The rna n-glycosidase activity of ricin a-chain. The characteristics of the enzymatic activity of ricin a-chain with ribosomes and with rrna. J. Biol. Chem..

[26] Montanaro L., Sperti S., Mattioli A., Testoni G., Stirpe F. (1975). Inhibition by ricin of protein synthesis in vitro. Inhibition of the binding of elongation factor 2 and of adenosine diphosphate-ribosylated elongation factor 2 to ribosomes. Biochem. J..

[27] Barbieri L., Valbonesi P., Bonora E., Gorini P., Bolognesi A., Stirpe F. (1997). Polynucleotide:Adenosine glycosidase activity of ribosome-inactivating proteins: Effect on dna, rna and poly(a). Nucleic Acids Res..

[28] Brigotti M., Alfieri R., Sestili P., Bonelli M., Petronini P.G., Guidarelli A., Barbieri L., Stirpe F., Sperti S. (2002). Damage to nuclear dna induced by shiga toxin 1 and ricin in human endothelial cells. FASEB J..

[29] Sikriwal D., Ghosh P., Batra J.K. (2008). Ribosome inactivating protein saporin induces apoptosis through mitochondrial cascade, independent of translation inhibition. Int. J. Biochem. Cell Biol..

[30] Das M.K., Sharma R.S., Mishra V. (2012). Induction of apoptosis by ribosome inactivating proteins: Importance of n-glycosidase activity. Appl. Biochem. Biotechnol..

[31] Gilabert-Oriol R., Weng A., Mallinckrodt B., Melzig M.F., Fuchs H., Thakur M. (2014). Immunotoxins constructed with ribosome-inactivating proteins and their enhancers: A lethal cocktail with tumor specific efficacy. Curr. Pharm. Des..

[32] Alewine C., Hassan R., Pastan I. (2015). Advances in anticancer immunotoxin therapy. Oncologist.

[33] Madhumathi J., Verma R.S. (2012). Therapeutic targets and recent advances in protein immunotoxins. Curr. Opin. Microbiol..

[34] Moolten F.L., Capparell N.J., Cooperband S.R. (1972). Antitumor effects of antibody-diphtheria toxin conjugates: Use of hapten-coated tumor cells as an antigenic target. J. Natl. Cancer Inst..

[35] Samagh B.S., Gregory K.F. (1972). Antibody to lactate dehydrogenase. V. Use as a carrier for introducing diphtheria toxin into mouse tumor cells. Biochim. Biophys. Acta.

[36] Lin J.Y., Tserng K.Y., Chen C.C., Lin L.T., Tung T.C. (1970). Abrin and ricin: New anti-tumour substances. Nature.

[37] Fodstad O., Olsnes S., Pihl A. (1976). Toxicity, distribution and elimination of the cancerostatic lectins abrin and ricin after parenteral injection into mice. Br. J. Cancer.

[38] Moolten F., Zajdel S., Cooperband S. (1976). Immunotherapy of experimental animal tumors with antitumor antibodies conjugated to diphtheria toxin or ricin. Ann. N. Y. Acad. Sci..

[39] Spitler L.E. (1986). Immunotoxin therapy of malignant melanoma. Med. Oncol. Tumor Pharmacother..

[40] Polito L., Djemil A., Bortolotti M. (2016). Plant toxin-based immunotoxins for cancer therapy: A short overview. Biomedicines.

[41] Youle R.J., Neville D.M. (1980). Anti-thy 1.2 monoclonal antibody linked to ricin is a potent cell-type-specific toxin. Proc. Natl. Acad. Sci. USA.

[42] Wawrzynczak E.J., Watson G.J., Cumber A.J., Henry R.V., Parnell G.D., Rieber E.P., Thorpe P.E. (1991). Blocked and non-blocked ricin immunotoxins against the cd4 antigen exhibit higher cytotoxic potency than a ricin a chain immunotoxin potentiated with ricin b chain or with a ricin b chain immunotoxin. Cancer Immunol. Immunother..

[43] Thorpe P.E., Brown A.N., Ross W.C., Cumber A.J., Detre S.I., Edwards D.C., Davies A.J., Stirpe F. (1981). Cytotoxicity acquired by conjugation of an anti-thy1.1 monoclonal antibody and the ribosome-inactivating protein, gelonin. Eur. J. Biochem..

[44] Ramakrishnan S., Houston L.L. (1984). Comparison of the selective cytotoxic effects of immunotoxins containing ricin a chain or pokeweed antiviral protein and anti-thy 1.1 monoclonal antibodies. Cancer Res..

[45] O’Hare M., Roberts L.M., Thorpe P.E., Watson G.J., Prior B., Lord J.M. (1987). Expression of ricin a chain in escherichia coli. FEBS Lett..

[46] Prieto I., Lappi D.A., Ong M., Matsunami R., Benatti L., Villares R., Soria M., Sarmientos P., Baird A. (1991). Expression and characterization of a basic fibroblast growth factor-saporin fusion protein in escherichia coli. Ann. N. Y. Acad. Sci..

[47] Rosenblum M.G., Kohr W.A., Beattie K.L., Beattie W.G., Marks W., Toman P.D., Cheung L. (1995). Amino acid sequence analysis, gene construction, cloning, and expression of gelonin, a toxin derived from gelonium multiflorum. J. Interferon Cytokine Res..

[48] Laurent G., Pris J., Farcet J.P., Carayon P., Blythman H., Casellas P., Poncelet P., Jansen F.K. (1986). Effects of therapy with t101 ricin a-chain immunotoxin in two leukemia patients. Blood.

[49] Spitler L.E., del Rio M., Khentigan A., Wedel N.I., Brophy N.A., Miller L.L., Harkonen W.S., Rosendorf L.L., Lee H.M., Mischak R.P. (1987). Therapy of patients with malignant melanoma using a monoclonal antimelanoma antibody-ricin a chain immunotoxin. Cancer Res..

[50] Falini B., Bolognesi A., Flenghi L., Tazzari P.L., Broe M.K., Stein H., Durkop H., Aversa F., Corneli P., Pizzolo G. (1992). Response of refractory hodgkin’s disease to monoclonal anti-cd30 immunotoxin. Lancet.

[51] LeMaistre C.F., Rosen S., Frankel A., Kornfeld S., Saria E., Meneghetti C., Drajesk J., Fishwild D., Scannon P., Byers V. (1991). Phase i trial of h65-rta immunoconjugate in patients with cutaneous t-cell lymphoma. Blood.

[52] Selvaggi K., Saria E.A., Schwartz R., Vlock D.R., Ackerman S., Wedel N., Kirkwood J.M., Jones H., Ernstoff M.S. (1993). Phase I/II study of murine monoclonal antibody-ricin a chain (xomazyme-mel) immunoconjugate plus cyclosporine a in patients with metastatic melanoma. J. Immunother. Emphasis Tumor Immunol..

[53] Baluna R., Vitetta E.S. (1997). Vascular leak syndrome: A side effect of immunotherapy. Immunopharmacology.

[54] Vitetta E.S. (2000). Immunotoxins and vascular leak syndrome. Cancer J..

[55] Ahmad A., Law K. (1988). Strategies for designing antibody-toxin conjugates. Trends Biotechnol..

[56] Shan L., Liu Y., Wang P. (2013). Recombinant immunotoxin therapy of solid tumors: Challenges and strategies. J. Basic Clin. Med..

[57] Hertler A.A., Frankel A.E. (1989). Immunotoxins: A clinical review of their use in the treatment of malignancies. J. Clin. Oncol..

[58] Harkonen S., Stoudemire J., Mischak R., Spitler L.E., Lopez H., Scannon P. (1987). Toxicity and immunogenicity of monoclonal antimelanoma antibody-ricin a chain immunotoxin in rats. Cancer Res..

[59] Li M., Liu Z.S., Liu X.L., Hui Q., Lu S.Y., Qu L.L., Li Y.S., Zhou Y., Ren H.L., Hu P. (2017). Clinical targeting recombinant immunotoxins for cancer therapy. OncoTargets Ther..

[60] Pastan I.H., Pai L.H., Brinkmann U., Fitzgerald D.J. (1995). Recombinant toxins: New therapeutic agents for cancer. Ann. N. Y. Acad. Sci..

[61] Weldon J.E., Skarzynski M., Therres J.A., Ostovitz J.R., Zhou H., Kreitman R.J., Pastan I. (2015). Designing the furin-cleavable linker in recombinant immunotoxins based on pseudomonas exotoxin a. Bioconj. Chem..

[62] Vallera D.A., Panoskaltsis-Mortari A., Blazar B.R. (1997). Renal dysfunction accounts for the dose limiting toxicity of dt390anti-cd3sfv, a potential new recombinant anti-gvhd immunotoxin. Protein Eng..

[63] Pennell C.A., Erickson H.A. (2002). Designing immunotoxins for cancer therapy. Immunol. Res..

[64] Pastan I., Hassan R., FitzGerald D.J., Kreitman R.J. (2007). Immunotoxin treatment of cancer. Annu. Rev. Med..

[65] Brinkmann U., Pai L.H., FitzGerald D.J., Willingham M., Pastan I. (1991). B3(fv)-pe38kdel, a single-chain immunotoxin that causes complete regression of a human carcinoma in mice. Proc. Natl. Acad. Sci. USA.

[66] Hamers-Casterman C., Atarhouch T., Muyldermans S., Robinson G., Hamers C., Songa E.B., Bendahman N., Hamers R. (1993). Naturally occurring antibodies devoid of light chains. Nature.

[67] Li T., Qi S., Unger M., Hou Y.N., Deng Q.W., Liu J., Lam C.M., Wang X.W., Xin D., Zhang P. (2016). Immuno-targeting the multifunctional cd38 using nanobody. Sci. Rep..

[68] Yu Y., Li J., Zhu X., Tang X., Bao Y., Sun X., Huang Y., Tian F., Liu X., Yang L. (2017). Humanized cd7 nanobody-based immunotoxins exhibit promising anti-t-cell acute lymphoblastic leukemia potential. Int. J. Nanomed..

[69] Tang J., Li J., Zhu X., Yu Y., Chen D., Yuan L., Gu Z., Zhang X., Qi L., Gong Z. (2016). Novel cd7-specific nanobody-based immunotoxins potently enhanced apoptosis of cd7-positive malignant cells. Oncotarget.

[70] Rosenblum M.G., Cheung L.H., Liu Y., Marks J.W. (2003). Design, expression, purification, and characterization, in vitro and in vivo, of an antimelanoma single-chain fv antibody fused to the toxin gelonin. Cancer Res..

[71] Zhou H., Ekmekcioglu S., Marks J.W., Mohamedali K.A., Asrani K., Phillips K.K., Brown S.A., Cheng E., Weiss M.B., Hittelman W.N. (2013). The tweak receptor fn14 is a novel therapeutic target in melanoma: Immunotoxins targeting fn14 receptor for malignant melanoma treatment. J. Investig. Dermatol..

[72] Woo J.H., Liu Y.Y., Mathias A., Stavrou S., Wang Z., Thompson J., Neville D.M. (2002). Gene optimization is necessary to express a bivalent anti-human anti-t cell immunotoxin in pichia pastoris. Protein Expr. Purif..

[73] Della Cristina P., Castagna M., Lombardi A., Barison E., Tagliabue G., Ceriotti A., Koutris I., Di Leandro L., Giansanti F., Vago R. (2015). Systematic comparison of single-chain fv antibody-fusion toxin constructs containing pseudomonas exotoxin a or saporin produced in different microbial expression systems. Microb. Cell Fact..

[74] Schindler J., Gajavelli S., Ravandi F., Shen Y., Parekh S., Braunchweig I., Barta S., Ghetie V., Vitetta E., Verma A. (2011). A phase i study of a combination of anti-cd19 and anti-cd22 immunotoxins (combotox) in adult patients with refractory b-lineage acute lymphoblastic leukaemia. Br. J. Haematol..

[75] Herrera L., Bostrom B., Gore L., Sandler E., Lew G., Schlegel P.G., Aquino V., Ghetie V., Vitetta E.S., Schindler J. (2009). A phase 1 study of combotox in pediatric patients with refractory b-lineage acute lymphoblastic leukemia. J. Pediatr. Hematol. Oncol..

[76] Furman R.R., Grossbard M.L., Johnson J.L., Pecora A.L., Cassileth P.A., Jung S.H., Peterson B.A., Nadler L.M., Freedman A., Bayer R.L. (2011). A phase iii study of anti-b4-blocked ricin as adjuvant therapy post-autologous bone marrow transplant: Calgb 9254. Leuk. Lymphoma.

[77] Gould B.J., Borowitz M.J., Groves E.S., Carter P.W., Anthony D., Weiner L.M., Frankel A.E. (1989). Phase i study of an anti-breast cancer immunotoxin by continuous infusion: Report of a targeted toxic effect not predicted by animal studies. J. Natl. Cancer Inst..

[78] Weiner L.M., O’Dwyer J., Kitson J., Comis R.L., Frankel A.E., Bauer R.J., Konrad M.S., Groves E.S. (1989). Phase I evaluation of an anti-breast carcinoma monoclonal antibody 260f9-recombinant ricin a chain immunoconjugate. Cancer Res..

[79] Borthakur G., Rosenblum M.G., Talpaz M., Daver N., Ravandi F., Faderl S., Freireich E.J., Kadia T., Garcia-Manero G., Kantarjian H. (2013). Phase 1 study of an anti-cd33 immunotoxin, humanized monoclonal antibody m195 conjugated to recombinant gelonin (hum-195/rgel), in patients with advanced myeloid malignancies. Haematologica.

[80] Turturro F. (2007). Denileukin diftitox: A biotherapeutic paradigm shift in the treatment of lymphoid-derived disorders. Expert Rev. Anticancer Ther..

[81] Hayashida K. (2013). Substance p-saporin for bone cancer pain in dogs: Can man’s best friend solve the lost in translation problem in analgesic development?. Anesthesiology.

[82] Brown D.C., Agnello K. (2013). Intrathecal substance p-saporin in the dog: Efficacy in bone cancer pain. Anesthesiology.

[83] Higgins S.C., Fillmore H.L., Ashkan K., Butt A.M., Pilkington G.J. (2015). Dual targeting ng2 and gd3a using mab-zap immunotoxin results in reduced glioma cell viability in vitro. Anticancer Res..

[84] Vallera D.A., Todhunter D.A., Kuroki D.W., Shu Y., Sicheneder A., Chen H. (2005). A bispecific recombinant immunotoxin, dt2219, targeting human cd19 and cd22 receptors in a mouse xenograft model of b-cell leukemia/lymphoma. Clin. Cancer Res..

[85] Vallera D.A., Oh S., Chen H., Shu Y., Frankel A.E. (2010). Bioengineering a unique deimmunized bispecific targeted toxin that simultaneously recognizes human cd22 and cd19 receptors in a mouse model of b-cell metastases. Mol. Cancer Ther..

[86] Fidias P., Grossbard M., Lynch T.J. (2002). A phase ii study of the immunotoxin n901-blocked ricin in small-cell lung cancer. Clin. Lung Cancer.

[87] Baluna R., Rizo J., Gordon B.E., Ghetie V., Vitetta E.S. (1999). Evidence for a structural motif in toxins and interleukin-2 that may be responsible for binding to endothelial cells and initiating vascular leak syndrome. Proc. Natl. Acad. Sci. USA.

[88] Smallshaw J.E., Ghetie V., Rizo J., Fulmer J.R., Trahan L.L., Ghetie M.A., Vitetta E.S. (2003). Genetic engineering of an immunotoxin to eliminate pulmonary vascular leak in mice. Nat. Biotechnol..

[89] Pastan I., Hassan R., Fitzgerald D.J., Kreitman R.J. (2006). Immunotoxin therapy of cancer. Nat. Rev. Cancer.

[90] Pirie C.M., Hackel B.J., Rosenblum M.G., Wittrup K.D. (2011). Convergent potency of internalized gelonin immunotoxins across varied cell lines, antigens, and targeting moieties. J. Biol. Chem..

[91] Pirie C.M., Liu D.V., Wittrup K.D. (2013). Targeted cytolysins synergistically potentiate cytoplasmic delivery of gelonin immunotoxin. Mol. Cancer Ther..

[92] Fuchs H., Bachran D., Panjideh H., Schellmann N., Weng A., Melzig M.F., Sutherland M., Bachran C. (2009). Saponins as tool for improved targeted tumor therapies. Curr. Drug Targets.

[93] Nakase I., Kobayashi S., Futaki S. (2010). Endosome-disruptive peptides for improving cytosolic delivery of bioactive macromolecules. Biopolymers.

[94] Berg K., Folini M., Prasmickaite L., Selbo P.K., Bonsted A., Engesaeter B.O., Zaffaroni N., Weyergang A., Dietze A., Maelandsmo G.M. (2007). Photochemical internalization: A new tool for drug delivery. Curr. Pharm. Biotechnol..

[95] Weyergang A., Selbo P.K., Berstad M.E., Bostad M., Berg K. (2011). Photochemical internalization of tumor-targeted protein toxins. Lasers Surg. Med..

[96] Bostad M., Olsen C.E., Peng Q., Berg K., Hogset A., Selbo P.K. (2015). Light-controlled endosomal escape of the novel cd133-targeting immunotoxin ac133-saporin by photochemical internalization—A minimally invasive cancer stem cell-targeting strategy. J. Control. Release.

[97] Queen C., Schneider W.P., Selick H.E., Payne P.W., Landolfi N.F., Duncan J.F., Avdalovic N.M., Levitt M., Junghans R.P., Waldmann T.A. (1989). A humanized antibody that binds to the interleukin 2 receptor. Proc. Natl. Acad. Sci. USA.

[98] Molineux G. (2002). Pegylation: Engineering improved pharmaceuticals for enhanced therapy. Cancer Treat. Rev..

[99] Tsutsumi Y., Onda M., Nagata S., Lee B., Kreitman R.J., Pastan I. (2000). Site-specific chemical modification with polyethylene glycol of recombinant immunotoxin anti-tac(fv)-pe38 (lmb-2) improves antitumor activity and reduces animal toxicity and immunogenicity. Proc. Natl. Acad. Sci. USA.

[100] Cheung L.H., Marks J.W., Rosenblum M.G. (2004). Development of “designer toxins” with reduced antigenicity and size. Proc. Amer. Assoc. Cancer Res..

[101] Cizeau J., Grenkow D.M., Brown J.G., Entwistle J., MacDonald G.C. (2009). Engineering and biological characterization of vb6–845, an anti-epcam immunotoxin containing a t-cell epitope-depleted variant of the plant toxin bouganin. J. Immunother..

[102] Entwistle J., Brown J.G., Chooniedass S., Cizeau J., MacDonald G.C. (2012). Preclinical evaluation of vb6–845: An anti-epcam immunotoxin with reduced immunogenic potential. Cancer Biother. Radiopharm..

[103] Liu W., Onda M., Lee B., Kreitman R.J., Hassan R., Xiang L., Pastan I. (2012). Recombinant immunotoxin engineered for low immunogenicity and antigenicity by identifying and silencing human b-cell epitopes. Proc. Natl. Acad. Sci. USA.

[104] Tachtsidis A., McInnes L.M., Jacobsen N., Thompson E.W., Saunders C.M. (2016). Minimal residual disease in breast cancer: An overview of circulating and disseminated tumour cells. Clin. Exp. Metastasis.

[105] Sarosdy M.F., Hutzler D.H., Yee D., von Hoff D.D. (1993). In vitro sensitivity testing of human bladder cancers and cell lines to tp-40, a hybrid protein with selective targeting and cytotoxicity. J. Urol..

[106] Battelli M.G., Polito L., Bolognesi A., Lafleur L., Fradet Y., Stirpe F. (1996). Toxicity of ribosome-inactivating proteins-containing immunotoxins to a human bladder carcinoma cell line. Int. J. Cancer.

[107] Li C., Yan R., Yang Z., Wang H., Zhang R., Chen H., Wang J. (2017). Bcmab1-ra, a novel immunotoxin that bcmab1 antibody coupled to ricin a chain, can eliminate bladder tumor. Oncotarget.

[108] Dalken B., Giesubel U., Knauer S.K., Wels W.S. (2006). Targeted induction of apoptosis by chimeric granzyme b fusion proteins carrying antibody and growth factor domains for cell recognition. Cell Death Differ..

[109] Rybak S.M., Arndt M.A., Schirrmann T., Dubel S., Krauss J. (2009). Ribonucleases and immunornases as anticancer drugs. Curr. Pharm. Des..

[110] Mathew M., Verma R.S. (2009). Humanized immunotoxins: A new generation of immunotoxins for targeted cancer therapy. Cancer Sci..

